# Complete genome sequences of a Canadian strain of enteroaggregative *Escherichia coli* (EAEC) with multiple metals and antimicrobial resistance genes isolated from municipal waste-activated sludge

**DOI:** 10.1128/mra.01242-23

**Published:** 2024-01-31

**Authors:** Mingsong Kang, Philippe Charron, Emily Hoover, Hongsheng Huang

**Affiliations:** 1 Canadian Food Inspection Agency, Ottawa Laboratory - Fallowfield, Ottawa, Ontario, Canada; Wellesley College Department of Biological Sciences, Wellesley, Massachusetts, USA

**Keywords:** enteroaggregative *Escherichia coli*, whole-genome sequencing, waste-activated sludge, multiple metals and antimicrobial resistance genes

## Abstract

Enteroaggregative *Escherichia coli* (EAEC) is an emerging food-borne pathogen causing acute or persistent diarrhea in humans. Here, we report the complete genome sequence of a strain of EAEC with multiple metals and antimicrobial resistance genes isolated from a waste-activated sludge collected from a Canadian municipal wastewater treatment plant.

## ANNOUNCEMENT

Enteroaggregative *Escherichia coli* (EAEC) causes diarrhea in humans and is responsible for both acute and persistent diarrhea worldwide (1). The current study reports the genome sequence of an EAEC strain (HH35) isolated from a waste-activated sludge collected from a Canadian municipal wastewater treatment plant in 2010. The strain was isolated by enrichment in lauryl sulfate tryptose broth (35°C, 24 hours) and then in *E. coli* broth (45°C, 24 hours), and isolation using Levine’s eosin methylene blue agar (35°C, 24 hours) (2). It was identified as EAEC, based on the presence of the *aggR* gene and a complete AAF/V gene cluster within its genome (3).

Genomic DNA (gDNA) extract, used for both sequencing procedures below, was prepared from an overnight culture grown from a single colony in Tryptic Soy Broth using NanoBind CBB Kit (PacBio, US), followed by treatment with Short Read Eliminator XS (PacBio, US), according to the manufacturer’s instructions. Illumina sequencing was conducted by library preparation using Illumina DNA Prep Kit (Illumina, US), and sequencing on the MiSeq platform (Illumina, US) using MiSeq reagent kit v3 with a total of 598,140 paired-end (300 bp) reads generated, followed by filtration and trimming with Fastp v.0.23.2 (4). Nanopore sequencing was performed by MinION library preparation using a ligation sequencing gDNA-native barcoding kit (SQK-NBD112.24) (Oxford Nanopore Technologies, UK) without shearing, and sequencing using an FLO-MIN112 (R10.4.1) flow cell on a MinION Mk1B device. A total of 2,191,450 reads (N50 of 2,128 bp) were obtained, followed by base-calling using Guppy v6.1.2, trimming using Porechop v0.2.4, and filtering using NanoFilt v2.8.0 (5). Assembly of MinION reads was performed using flye v2.9.1 (6) and polished with medaka v1.7.2 (https://github.com/nanoporetech/medaka), followed by short-read polishing using NextPolish v1.4.1 (7), ntEdit v1.3.5 (8), and Polypolish v0.5.0 (9). The circularity of the genome and genome rotation using *dnaA* as the starting point was determined by Circlator v1.5.5 (10). The sequencing coverage depth (474×) was determined and assessed using Samtools v1.13 (11). Gene predictions and annotations were performed using NCBI Prokaryotic Genome Annotation Pipeline v6.4 (12). Metal, acid, virulence, and antimicrobial resistance genes were identified using AMRFinderPlus v3.11.18 with database v2023-08-08.2 (13). The plasmids were identified by PlasmidFinder v2.0.1 with database v2023-01-18 (14), and prophage sequences were analyzed using the PHASTER web server (15). Its serotype was identified using ECTyper v1.0.0 (16), and pathogenicity was predicted using PathogenFinder v1.1 (17). Default parameters of bioinformatics tools were used except where otherwise noted.

The HH35 isolate was predicted as serotype O99:H10 EAEC. Its genome contains a single chromosome with one plasmid. Table 1 demonstrates detailed information for total length, chromosome size, GC%, protein count, prophage, antibiotics, heavy metal, acid resistance, and virulence genes. On average, the median total length, CDS, and GC% of *E. coli* genome assemblies in GenBank are similar to those of this *E. coli* strain (Table 1).

**TABLE 1:** Genomic characteristics of the *EAEC* strain (HH35) isolated from a municipal waste-activated sludge

Strain ID	Contigs	Plasmid	Total length (Mb)	Chromosome size (bp)	GC%	Protein counts	Genes related to AMR^*a*^	Genes related to metal-resistance^*a*^	Genes related to acid-resistance^*a*^	Virulence genes^*a*^	Intact prophage
HH35	2	1	5.02	4,916,664	50.7%	4572	10 *mdtM blaEC emrD acrF dfrA17* *tet(A) aph (6)-Id aph(3'')-Ib sul2* *blaTEM-1*	3 *fieF arsC arsR*	2 *asr ariR*	13 *fdeC espX1* *capU aaiC pic aatA aar aap aggR agg5A agg3B agg3C agg3D*	3
Median GenBank sequences^*b*^			5.10		50.6%	4727					

^*a*^ Antimicrobial, heavy metal, and acid resistance, and virulence genes were predicted by AMRFinderPlus.

^*b*^ Data summarized on 11–14-2023 using 36,469 *E. coli* genome assemblies available on GenBank.

## Data Availability

The genome and plasmid sequences of strain HH35 have been deposited at GenBank under accession numbers CP136993.2 and CP136994.1. MinION and MiSeq raw data are available in the NCBI Sequence Read Archive through accession numbers SRR26967109 and SRR26407666, respectively.

## References

[1] Kaur P, Chakraborti A, Asea A. 2010. Enteroaggregative Escherichia coli: an emerging enteric food borne pathogen. Interdiscip Perspect Infect Dis 2010:254159. doi:10.1155/2010/254159 20300577 PMC2837894

[2] Kang M, Chmara J, Naushad S, Huang H. 2021. Complete genome sequence of a Canadian strain of Raoultella planticola with metal and antimicrobial resistance genes. Microbiol Resour Announc 10:e0041521. doi:10.1128/MRA.00415-21 34165338 PMC8223810

[3] Boisen N, Østerlund MT, Joensen KG, Santiago AE, Mandomando I, Cravioto A, Chattaway MA, Gonyar LA, Overballe-Petersen S, Stine OC, Rasko DA, Scheutz F, Nataro JP. 2020. Redefining enteroaggregative Escherichia coli (EAEC): genomic characterization of epidemiological EAEC strains. PLoS Negl Trop Dis 14:e0008613. doi:10.1371/journal.pntd.0008613 32898134 PMC7500659

[4] Chen S, Zhou Y, Chen Y, Gu J. 2018. Fastp: an ultra-fast all-in-one FASTQ preprocessor. Bioinformatics 34:i884–i890. doi:10.1093/bioinformatics/bty560 30423086 PMC6129281

[5] De Coster W, D’Hert S, Schultz DT, Cruts M, Van Broeckhoven C. 2018. NanoPack: visualizing and processing long-read sequencing data. Bioinformatics 34:2666–2669. doi:10.1093/bioinformatics/bty149 29547981 PMC6061794

[6] Freire B, Ladra S, Parama JR. 2022. Memory-efficient assembly using flye. IEEE/ACM Trans Comput Biol Bioinform 19:3564–3577. doi:10.1109/TCBB.2021.3108843 34469305

[7] Hu J, Fan J, Sun Z, Liu S. 2020. NextPolish: a fast and efficient genome polishing tool for long-read assembly. Bioinformatics 36:2253–2255. doi:10.1093/bioinformatics/btz891 31778144

[8] Warren RL, Coombe L, Mohamadi H, Zhang J, Jaquish B, Isabel N, Jones SJM, Bousquet J, Bohlmann J, Birol I. 2019. ntEdit: scalable genome sequence polishing. Bioinformatics 35:4430–4432. doi:10.1093/bioinformatics/btz400 31095290 PMC6821332

[9] Wick RR, Holt KE. 2022. Polypolish: short-read polishing of long-read bacterial genome assemblies. PLoS Comput Biol 18:e1009802. doi:10.1371/journal.pcbi.1009802 35073327 PMC8812927

[10] Hunt M, Silva ND, Otto TD, Parkhill J, Keane JA, Harris SR. 2015. Circlator: automated circularization of genome assemblies using long sequencing reads. Genome Biol 16:294. doi:10.1186/s13059-015-0849-0 26714481 PMC4699355

[11] Li H, Handsaker B, Wysoker A, Fennell T, Ruan J, Homer N, Marth G, Abecasis G, Durbin R, Genome Project Data Processing S. 2009. The sequence alignment/map format and SAMtools. Bioinformatics 25:2078–2079. doi:10.1093/bioinformatics/btp352 19505943 PMC2723002

[12] Tatusova T, DiCuccio M, Badretdin A, Chetvernin V, Nawrocki EP, Zaslavsky L, Lomsadze A, Pruitt KD, Borodovsky M, Ostell J. 2016. NCBI prokaryotic genome annotation pipeline. Nucleic Acids Res 44:6614–6624. doi:10.1093/nar/gkw569 27342282 PMC5001611

[13] Feldgarden M, Brover V, Gonzalez-Escalona N, Frye JG, Haendiges J, Haft DH, Hoffmann M, Pettengill JB, Prasad AB, Tillman GE, Tyson GH, Klimke W. 2021. AMRFinderPlus and the reference gene catalog facilitate examination of the genomic links among antimicrobial resistance, stress response, and virulence. Sci Rep 11:12728. doi:10.1038/s41598-021-91456-0 34135355 PMC8208984

[14] Carattoli A, Hasman H. 2020. PlasmidFinder and in silico pMLST: identification and typing of plasmid replicons in whole-genome sequencing (WGS). Methods Mol Biol 2075:285–294. doi:10.1007/978-1-4939-9877-7_20 31584170

[15] Arndt D, Grant JR, Marcu A, Sajed T, Pon A, Liang Y, Wishart DS. 2016. PHASTER: a better, faster version of the PHAST phage search tool. Nucleic Acids Res 44:W16–W21. doi:10.1093/nar/gkw387 27141966 PMC4987931

[16] Joensen KG, Tetzschner AMM, Iguchi A, Aarestrup FM, Scheutz F. 2015. Rapid and easy in silico serotyping of Escherichia coli isolates by use of whole-genome sequencing data. J Clin Microbiol 53:2410–2426. doi:10.1128/JCM.00008-15 25972421 PMC4508402

[17] Cosentino S, Voldby Larsen M, Møller Aarestrup F, Lund O. 2013. PathogenFinder--distinguishing friend from foe using bacterial whole genome sequence data. PLoS One 8:e77302. doi:10.1371/journal.pone.0077302 24204795 PMC3810466

